# Esophageal Squamous Cell Carcinoma Presenting with* Streptococcus intermedius* Cerebral Abscess

**DOI:** 10.1155/2017/5819676

**Published:** 2017-08-15

**Authors:** Rabih Nayfe, Mustafa S. Ascha, Esther H. Rehmus

**Affiliations:** ^1^Department of Geriatrics, UT Southwestern Medical Center, Dallas, TX, USA; ^2^Department of Physiology and Biophysics, Case Western Reserve University, Cleveland, OH, USA; ^3^McDowell Cancer Institute, Cleveland Clinic Akron General, Akron, OH, USA

## Abstract

**Background:**

Cerebral abscess is caused by inoculation of an organism into the brain parenchyma from a site distant from the central nervous system.* Streptococcus intermedius (S. intermedius)* is a commensal organism that is normally present in the aerodigestive tract and was reported to be the cause of brain abscesses after esophageal dilatation or upper endoscopy.

**Case Presentation:**

We report the case of a 53-year-old female who presented with hematemesis and melena followed by left-sided weakness. Initially, her hemiplegia was found to be secondary to a right thalamic brain abscess caused by* S. intermedius*. Investigations led to the diagnosis of a mid-esophageal squamous cell carcinoma. We hypothesize that the cause of the abscess with this bacterium that naturally resides in the digestive tract and oral cavity is secondary to hematogenous spread from breach in the mucosal integrity from ulceration due to the cancer.

**Conclusion:**

To our knowledge, our case is the first in the literature to describe a brain abscess caused by* S. intermedius* in association with a previously undiagnosed esophageal squamous cell carcinoma without any prior esophageal intervention.

## 1. Background

While early detection using CT scans and subsequent antibiotic treatment have helped lower the mortality associated with brain abscess, the case fatality rate remains around 20% [1]. One of the greatest risk factors is an immunocompromised state, but immunocompetent patients are also at risk.

Brain abscesses occur when an organism seeds the brain parenchyma, leading to purulence and inflammation in one or more localized regions [2]. Three mechanisms for the development of intracerebral abscesses have been proposed: (1) direct extension from contiguous structures such as the teeth, middle ear, sinuses, or mastoid via venous circulation; (2) hematogenous spread from other sites such as the GI tract, heart, or lungs; and (3) following neurosurgical procedures or penetrating trauma to the skull [3]. Here, we focus on hematogenous spread of what is normally a part of GI flora.

*Streptococcus intermedius (S. intermedius)* is a commensal organism that is present in various mucosal sites, including the oral cavity, gastrointestinal tract, and genitourinary system. Its role in the development of invasive suppurative infections at various sites, including brain abscesses, has been described in multiple reports [4].

More specifically, brain abscess secondary to esophageal intervention has been reported after dilatation of benign esophageal strictures [5–7]. In fact, the risk of bacteremia as a result of GI tract stressors is great enough to warrant recommendations for antibiotic prophylaxis in some endoscopic procedures [8]. We report a case of undiagnosed esophageal squamous cell carcinoma associated with a brain abscess caused by* S. intermedius,* without any previous GI intervention.

## 2. Case Report

A 53-year-old Caucasian female presented to emergency medicine for abrupt onset of hematemesis and melena and was discharged after she was determined medically stable. The following day she returned with left lower extremity weakness, headache, and fever of 102°F, without recurrence of hematemesis or melena.

There was no significant past medical history, but the patient did have a 60-pack-year smoking history, continuing to smoke 1.5 packs of cigarettes daily. The patient also had a 13-year history of alcohol dependence at 12 drinks per day, but reported quitting drinking 4 weeks prior to presentation. Clinical examination was significant for left-sided upper and lower extremity weakness. Admission investigations revealed normocytic anemia with hemoglobin at 9.1 g/dL (reference range 11.7–14.7 g/dL), low albumin at 1.9 g/dl (reference range 3.4–5.0 g/dL), and low total protein at 4.8 g/dL (reference range 6.4–8.2 g/dL). Her coagulation profile, electrolytes, and liver function were within the normal limits.

**Figure 1:**
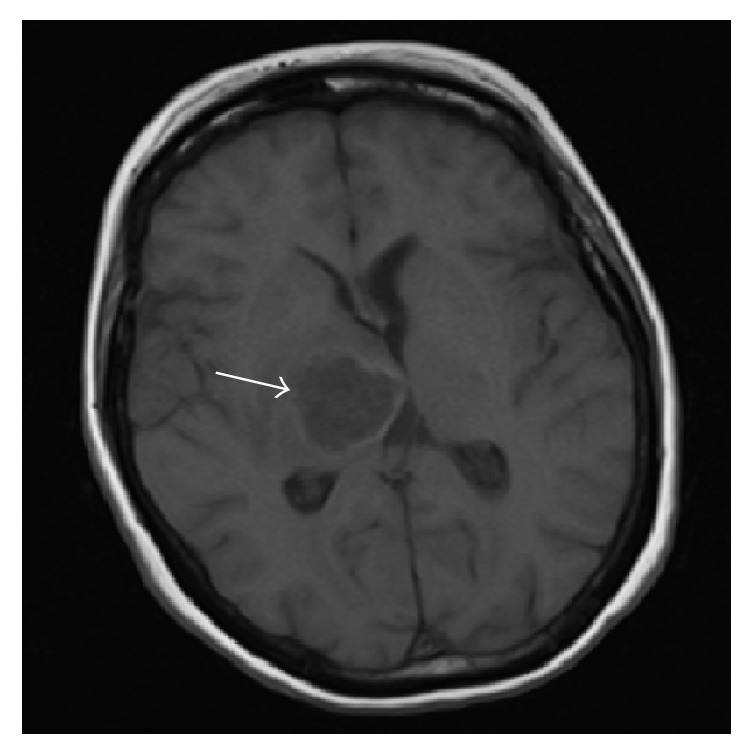
MRI brain with contrast showing intra-axial centered abscess (arrow) in the right thalamic region with associated right to left midline shift and downward herniation.

Noncontrast brain CT scan revealed an intra-axial lesion centered in the right thalamic region measuring 3.5 cm in maximal diameter with associated midline shift and downward herniation, most likely representing an abscess. Subsequent MRI of the brain with and without contrast confirmed CT scan findings, showing a right thalamic ring-enhancing lesion with vasogenic edema, right to left midline shift, and mild downward herniation (Figure 1).

The patient was admitted to the neurosurgical intensive care unit (NSICU) and then the operating room for further management. The thalamic abscess was drained under stereotactic guidance, and an intracavitary drain was placed. Operative findings included frank purulence from the abscess cavity. She remained intubated and sedated and was returned to the NSICU in stable condition. Cultures from the abscess grew* S. intermedius*, and, based on bacterial sensitivities, she was maintained on vancomycin, ceftriaxone, and metronidazole. No malignant cells were identified in abscess fluid.

**Figure 2:**
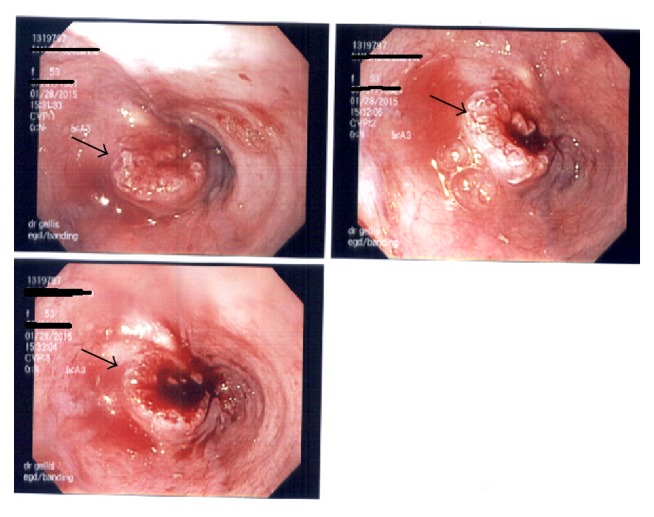
Images from an esophagogastroduodenoscopy showing a large obstructing mid-esophageal mass (arrows) at 25 cm.

**Figure 3:**
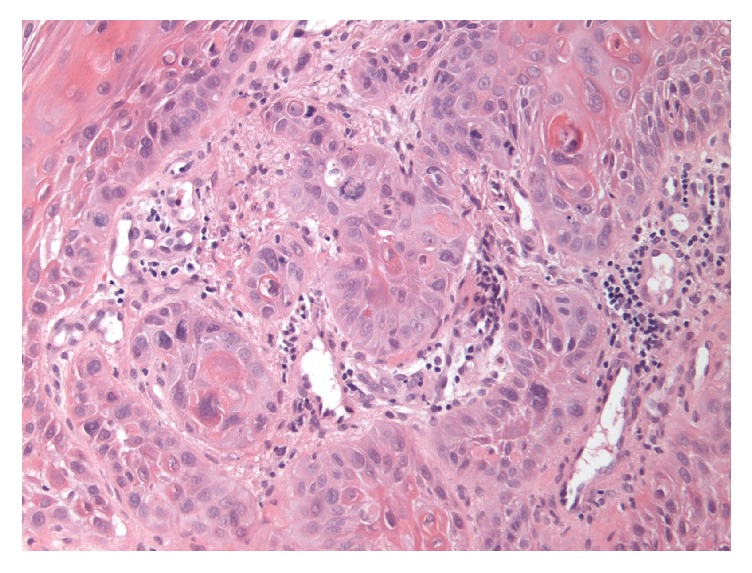
Biopsy of esophageal mass showing invasive squamous cell carcinoma.

Given a history of hematemesis, melena, anemia, and chronic alcohol abuse, an esophagogastroduodenoscopy (EGD) was scheduled to rule out possible causes of upper gastrointestinal bleed. EGD showed a large obstructing mid-esophageal mass at 25 cm, beyond which the scope could not be advanced despite moderate pressure (Figure 2). Biopsies of the esophageal mass revealed invasive squamous cell carcinoma (Figure 3).

Subsequent staging CT scan of the chest/abdomen/pelvis showed the esophageal mass measured up to 38 × 26 mm in transverse and anteroposterior dimensions, respectively, causing severe luminal compromise with proximal esophageal dilatation. CT scan findings also included mediastinal and right hilar lymphadenopathy. There was no metastatic disease within the abdomen and pelvis and no signs of extracerebral infection.

Postoperative recovery was uneventful; the patient was weaned off mechanical ventilation and successfully extubated on day four after surgery. She was able to follow simple and complex commands with normal motor function on her right side, but paralysis remained in the left upper and lower extremities. There was progressive improvement in sensory and motor function with therapy. After sufficient drainage, the intracavitary drain was removed.

Total parental nutrition was initially used due to complaints of dysphagia. General surgery was then consulted for palliative esophageal stent placement; this was well-tolerated and oral nutrition was resumed.

The patient was not considered a candidate for any chemotherapy or radiation out of concern regarding infection exacerbation. Carcinoma treatment, then, would be reevaluated after the patient completed a 6-week prescription of antibiotics.

## 3. Discussion

To the best of our knowledge, this is the first report of a* S. intermedius* induced brain abscess that is associated with an esophageal squamous cell carcinoma. Though there is significant evidence that therapeutic breach of esophageal mucosa during endoscopy can lead to bacteremia, esophageal squamous cell carcinoma leading to bacteremia and subsequent brain abscess appears novel.

Brain abscesses are caused by inoculation of the brain parenchyma. While a greater proportion of these abscesses involve inoculation from contiguous sites such as the mastoid or retrograde travel along emissary veins, some infections arrive from more distant sites. In a review of 102 cases, Helweg-Larsen et al. found that 28% of brain abscesses were a result of hematogenous spread from distant sites such as the heart, lungs, or gastrointestinal tract [9]. Even though the vast majority of bacteremic events do not result in brain abscess or CNS infection, the severity of potential outcomes makes such events noteworthy.

Hematogenous spread from the esophagus was suspected because* S. intermedius* grew on abscess fluid culture but is a commensal organism normally present in the oral cavity and airways. Further, previous work shows that* S. intermedius* can cause brain abscess in patients who do not have cancer but have recently had esophageal intervention [4].

*S. intermedius* as a cause of brain abscess was first reported in 1975, after which it was discovered that risk factors for brain abscesses due to this organism may include mucosal infection, pneumonia, liver abscess, alcohol abuse, and diabetes [4, 10]. Our patient had a longstanding history of alcohol abuse, which is also a major risk factor for cancers of the upper gastrointestinal tract. We suspect that alcoholism was conducive to the observed brain abscess, particularly given the immunosuppressive effects associated with alcohol consumption [11].

*Streptococcus anginosus*, which together with* S. intermedius* and* S. constellatus* form the “*Streptococcus anginosus* group,” has recently been associated with carcinogenesis in the aerodigestive tract. Morita et al. investigated this association, comparing the ratio of* S. anginosus* to other oral bacteria in the saliva of 38 alcoholics, 22 healthy people, 41 esophageal cancer patients, 32 gastritis patients, and 24 periodontitis patients. They reported very high levels of* S. anginosus* in saliva of people with alcoholism compared to the other groups. Though not statistically significant, the authors also found that the average ratio of* S. anginosus* to oral bacteria in the saliva of esophageal cancer patients was higher than that of healthy people. Each of these points helps explain the above patient's presentation, where likely high concentrations of* S. anginosus* species may have migrated from an esophageal cancer site in a patient with alcoholism [12].

In previously reported cases of brain abscess caused by* S. intermedius*, esophageal microperforation secondary to esophageal dilatation or other intervention likely opened the bloodstream to gut flora [13]. In the only other case of brain abscess secondary to* S. intermedius,* Hanna and Das report a patient with multiple abscesses and esophageal adenocarcinoma [2]. Our patient also presented with brain abscess, except with squamous cell carcinoma in place of adenocarcinoma. These different pathologies may indicate that it is the loss of mucosal integrity itself, rather than the underlying disease, that is associated with brain abscess.

Given the type of bacterium, the lack of endoscopy prior to diagnosis, and a prior report of a similar case, we expect that the brain abscess in this patient was a result of hematogenous bacterial spread across esophageal mucosa that was damaged by presence of squamous cell carcinoma.

## 4. Conclusion

Cerebral abscess is rare and life-threatening, and vigilant review of patient history could help identify cases such as the above. Our report of esophageal squamous cell carcinoma presenting with* S. intermedius*-associated cerebral abscess reminds us that if oral flora are isolated as pathogens from brain abscesses, a primary etiological reason in the gastrointestinal tract, especially the esophagus, should be looked for.
